# Mating compatibility and offspring traits evaluation among different strains of *Tenebrio molitor*

**DOI:** 10.1007/s11356-023-29116-1

**Published:** 2023-08-16

**Authors:** Christina Adamaki-Sotiraki, Christos I. Rumbos, Christos G. Athanassiou

**Affiliations:** Laboratory of Entomology and Agricultural Zoology, Department of Agriculture, Crop Production and Rural Environment, University of Thessaly, 38446 Volos, Greece

**Keywords:** Cross-breeding, Insect strains, Insects as food and feed, *Tenebrio molitor*, Inbred strains, Outbred strains

## Abstract

The fast-growing sector of insects for food and feed stimulates researchers and the industry to explore uncharted territories, such as insect breeding, to improve economically important insect fitness traits. The yellow mealworm, *Tenebrio molitor* L., is one of the most thoroughly studied insect species as food and feed. However, data on mating compatibility and the effect of cross-breeding between different strains on the performance and fitness of the hybrids are scarce. In the present study, we comparatively evaluated the mating compatibility between two *T. molitor* inbred strains (Greek and Italian) and their outbred strains, i.e., Italian (♀)-Greek (♂) and Greek (♀)-Italian (♂), as well as the performance of their hybrid offspring. Based on the results, there is good mating compatibility among adults of the strains tested. Offspring performance, quantified as larval survival and final larval weight, followed a similar pattern among the crossings examined. Even though differences were insignificant, the outbreeding of females of the Italian strain resulted in a higher cumulative number of eggs and hatching rate and higher offspring weight. The present study sheds light on the mating compatibility of different *T. molitor* strains and their hybrids' economically important life table characteristics to take the mass production of insects one step further.

## Introduction

Insect production as an alternative protein source has been gaining upscaling attention during the last decade, as the development of feed sources derived from insects is consistent with the principles of the circular economy (Van Huis and Oonincx 2017). The recent European regulations consider insects as promising solutions for livestock producers who are seeking new protein-rich and nutritious products as feed (EC 2017, 2021b, EFSA et al.^2021^). Moreover, the European Commission aims the promotion of the organic sector; thus, two aspiring framework policies have introduced recently. One of the ambitions included in these framework policies is targeting the reduction of the farmland being used for organic farming by at least 25% by 2030. This goal would be of utmost importance for developing the insect sector in Europe, helping the transition to more sustainable food systems (EC 2020, 2021a). The yellow mealworm, *Tenebrio molitor* L. (Coleoptera: Tenebrionidae), is one of the most widely produced and commercialized insect species used as feed for farmed animals, in terms of novel protein sources (Rumbos and Athanassiou 2021; Van Huis 2013). The utilization of its larvae has started with the pet food industry (Martin et al. 1976), while during the last few years, it has been considered an essential alternative protein and lipid source for aquaculture (EC 2017), livestock (EC 2021b) as well as human consumption (EFSA et al. 2021).

Based on the above, the insect sector has to meet the needs of the fast-growing insect protein demands. In this context, diet optimization, as well as the improvement of rearing conditions, have attracted vast attention (Heckmann et al. 2018; Morales-Ramos et al. 2020; Rumbos et al. 2020). Another factor that has also been investigated and may contribute to the up-scaling of mass production of insects is the strain effect in terms of larval performance and adult fitness (Adamaki-Sotiraki et al. 2022a, b; Rumbos et al. 2021; Urs and Hopkins 1973b, a). Based on the available literature, an aspect that has not investigated thoroughly yet concerning insects as food and feed is the effect of cross-breeding of strains derived from different geographical areas, in respect of adult mating compatibility as well as hybrids’ fitness traits, but also on the performance of the hybrid populations that will produced after successful mating.

The idea of cross-breeding is not new for the traditional crops, livestock, and insects' sector (e.g., sericulture, apiculture, and biological pest control), as it has been operated for thousands of years and thus is currently a fine-tuned methodology (Eriksson and Picard 2021; Lippman and Zamir 2007; Notter et al. 2013). However, the main objective behind the idea of cross-breeding is to produce hybrids with superior phenotypes relative to their inbred parents regarding traits such as growth rate, reproductive success, and yield. The specific procedure is known as heterosis, or hybrid vigor (Lippman and Zamir 2007). For example, concerning sericulture, for many years now, commercial varieties of the silkworm have been produced by the single cross, double cross, or three-way cross (Sohn and Ramirez 1999). In this way, silkworm breeders managed to produce varieties with optimized economic traits such as a higher percentage of cocoon shell, raw silk, and greater egg production (Reddy et al. 2010; Sohn and Ramirez 1999). Additionally, in apiculture protocols focusing on the selective mating of queens, specific trait selection and selective breeding are well established, aiming to minimize the depletion of genetic variation (Büchler et al. 2013; Uzunov et al. 2017). Moreover, concerning the mass production of natural enemies, it is necessary to maintain separate laboratory strains and cross them systematically to increase the fitness traits of the F1 generation (Van Lenteren et al. 2004).

For *T. molitor*, only one work dating back to the 1930s is available on its genetics and the effect of cross-breeding on specific insect characteristics; however, this mainly focused on crosses with different eye color genotypes (Schuurman 2013). More recently published data reveal that genetic material affects economically important life table characteristics, which may contribute to the upscaling of the overall productivity of mass-rearing facilities. Indicatively, (Urs and Hopkins 1973b, a) evaluated two different *T. molitor* strains and reported that the faster-developing strain was capable of more efficient nutrient utilization. (Rumbos et al. 2021) reported that specific strains of *T. molitor* perform better concerning larval survival, weight, feed utilization, and development time. Similarly, (Adamaki-Sotiraki et al. 2022a) support that growth performance varies among different *T. molitor* strains reared under dry conditions. Moreover, (Berggreen et al. 2018) urge that there is a strain effect also on adults’ fitness-related traits, while (Adamaki-Sotiraki et al. 2022b) also reported that specific strains performed better in terms of adult survival and fecundity. It is a rationale that different strains express some, but not all, the desired characteristics. Cross-breeding of different strains could be utilized to produce hybrids with optimal traits derived from different parental strains. Cross-breeding has successfully applied though in other insect species. Indicatively, in the sector of sericulture, researchers utilized divergent parental strains of the silkworm *Bombyx mori* (L.) (Lepidoptera: Bombycidae) and produced hybrids with better economic traits and technological characteristics (Bhargava et al. 1995; Doddaswamy et al. 2009; Reddy et al. 2010).

The studies mentioned above bring to the fore the value of cross-breeding among strains toward optimization of economically important traits aiming to upscale the profit in large-scale production industries. Therefore, investigation and understanding the contribution and association of various performance and fitness traits are essential for any program aiming to improve insect-rearing practices. Based on the above, the present study aims to investigate the mating compatibility (in terms of adult survival, egg production, and larval hatching rate) among adults of different *T. molitor* strains, as well as the effect of cross-breeding on important fitness traits of F1 hybrid larvae. For this purpose, we have selected specific strains of *T. molitor* of different geographical origins that have shown desirable characteristics in earlier experimentation (Adamaki-Sotiraki et al. 2022a, b; Rumbos et al. 2021).

## Materials and methods

Adult beetles and larvae of *T*. *molitor* derived from two different geographical areas, Greece and Italy, formed the materials for the present bioassays. The reason for the selection of the specific strains was the fact that they were reported to be among the strains that were previously shown to have superior performance in terms of adult fecundity, larval weight, and feed conversion efficiency (Adamaki-Sotiraki et al. 2022a; Rumbos et al. 2021). Rumbos et al (2021) have provided a detailed description of the traits of the strains used in the present study. The insects maintained at stable rearing conditions, i.e., continuous darkness, and 26 ± 0.5 °C, 50 ± 5% relative humidity, at the Laboratory of Entomology and Agricultural Zoology of the University of Thessaly. As insect feed, local wheat bran used, while agar provided as a moisture source.

### Experimental design

#### Bioassay I

In this bioassay, the parameters that were evaluated were adult survival, egg production and hatching rate. At first, a stereoscope (Stereomicroscope Leica MZ12, Spach Optics Inc, NY, United States) was used to separate the female and male pupae based on the differences in abdominal appendages (Arena and Defagó 2020; Wang et al. 2013). After sex determination, female and male pupae were kept separately until adult emergence. Newly-emerged, virgin dark-colored adults (7–14 days old, to ensure sexual maturity) used in the experiment (Jehan et al. 2020). After that, four groups of five male and five female adult beetles were weighted and placed in the oviposition boxes for each one of the cross-breeding combinations between the two strains (two inbred strains and two outbred strains). In total, 24 oviposition boxes were utilized. As oviposition boxes, two cylindrical plastic vials (8.8 cm height, and 7.5 cm diameter) stuck inside one another. The bottom of the upper vial was removed and replaced by a circular plastic mesh (2 × 2 mm hole diameter) to permit egg oviposition and avoid egg cannibalism by the adults. The bottom vial was filled with white flour as an oviposition substrate, up to 5 mm above the mesh, to ensure that adult beetles have an adequate feed. Agar cubes (20 g/L, 1 × 1x1 cm) provided to beetles as a moisture source ad libitum (Deruytter et al. 2021). According to Gerber and Sabourin (1984) beetles mated and oviposited for seven days. After this period of time, a 600 μm opening sieve was utilized in order to sieve the flour and collect and count the laid eggs, whereas the alive and dead beetles in each vial were also counted to determine adult survival. Alive adults were then transferred to new vials. The exact process was repeated eight times with the same adults, for a total period of 56 days. The duration of the bioassay was 56 days as it has been reported that females may be fertile for up to two months (Berggreen et al. 2018). Collected eggs were transferred in new experimental units (vials) together with 1 g of wheat bran, which served as substrate for the newly-emerged larvae to feed. After a period of 8 days, the number of hatched larvae was estimated, by counting the larvae. For each treatment, six replicates were carried out.

#### Bioassay II

In this series of bioassays, the larval weight, survival and development time of the F1 generation larvae produced during the first and the eighth week of bioassay I were recorded. Larvae produced during the first week were separated into groups of 50 individuals at six replicates, while, due to lower availability, larvae produced during the eighth week divided into groups of 15 individuals at four replicates. Larvae produced during the first week were kept separately from the larvae produced during the eighth week. The bioassay was carried out in plastic cylindrical vials as above, and the newly-emerged larvae were provided with 4 g of local wheat bran and agar cubes (1 × 1x1 cm) as a moisture source. In total, 40 vials were utilized. Every two weeks, for each vial, larvae were isolated from the substrate utilizing sieves with openings ranging from 500 μm to 1 mm, depending on the age of the larvae, in order to sieve the feed, collect the larvae, and determine their total weight, and survival. The larval weight was recorded utilizing a precision balance (EQUINOX, Adam Equipment Inc Fox Hollow Road, Oxford, USA). The same procedure repeated up to one week after the appearance of the first pupa. The development time from newly-hatched larvae to first pupae appearance was also calculated. Feed and moisture sources provided ad libitum based on visual observations. Trials were performed in controlled rearing conditions, as previously described.

#### Data analysis

To determine the larval hatching rate at each evaluation point, the number of emerged larvae divided by the respective number of eggs. In order to calculate the cumulative hatching rate, the cumulative number of emerged larvae divided by the cumulative number of eggs for each tested strain and at each point of evaluation. Normal distribution tested by the Shapiro–Wilk test while homogeneity/homoscedasticity of variances tested using Levene's test checked for final adult survival, the cumulative number of eggs per adult, cumulative hatching rate, final larval weight and survival rate, as well as larval development time. Since the assumptions needed for parametric analysis met for the final adult survival, the cumulative number of eggs, cumulative hatchability, final larval weight, and survival rate, data were submitted to ANOVA to determine differences among strains, as well as differences between larvae produced from adults of different age for the same strain (P < 0.05). Concerning the adult survival, the number of eggs per adult, and the hatching rate of the larvae, a mixed effect modeling fitted to be performed due to the longitudinal nature of the data. As a consequence, a linear mixed effect model applied to investigate the interaction of different factors [strain and time (days)] on adult survival, on larval hatching rate and on the number of eggs per adult (Shek and Ma 2011). The linear mixed model used to compare the time-series data of all inbred and outbred strains in a holistic way and to supply a direct statistical comparison among inbred and outbred strains in terms of adult survival, larval hatching rate, and number of eggs per adult during the whole experimental period of time. The Kaplan–Meier method was applied to analyze the development time of larvae produced during the first and the eighth week after adults' mating, and afterwards, Mantel-Cox test used to detect contrasts between treatments.

## Results

### Bioassay I

**Fig. 1:**
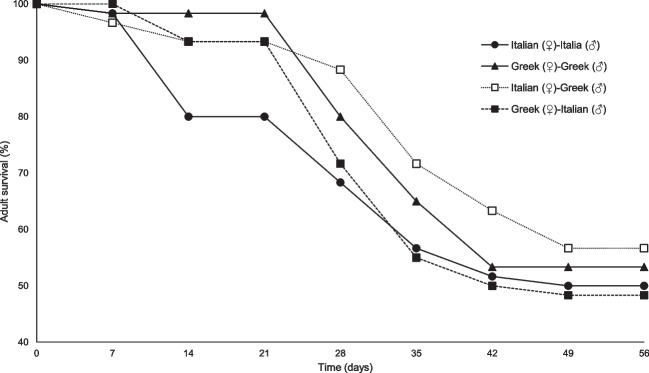
Survival rate (%) of adults of two inbred strains (Greek and Italian) and their outbred strains (Italian (♀)-Greek (♂) and Greek (♀)-Italian (♂)) of *Tenebrio molitor* (n = 6)

For all inbred and outbred *T. molitor* strains, adult survival decreased over time. While no significant differences detected concerning the final survival (56 days), the linear mixed model analysis revealed that both strain (F = 7.5, P < 0.001) and time (F = 62.2, P < 0.001) significantly affected adult survival over time (Fig. 1; Table 1). The first sharp reduction in adult survival occurred 28 days after the onset of the bioassay for the inbred Italian strain (68% ± 17%) and the outbred Greek (♀)-Italian (♂) strain (72% ± 13%). For the same time interval, adult survival ranged between 80 and 88% for the outbred Italian (♀)-Greek (♂) strain and the inbred Greek strain, respectively. During the termination of the bioassay (56 days), the rate of adult survival ranged between 50% ± 16% and 57% ± 10% for all inbred and outbred strains. The highest survival rate recorded for the outbred Italian (♀)-Greek (♂) strain and the lowest for the outbred Greek (♀)-Italian (♂) strain.

**Table 1:** Linear mixed model effect for adult survival (%), number of eggs per adult, and hatching rate (%) of two inbred strains (Greek and Italian) and their outbred strains (Italian (♀)-Greek (♂) and Greek (♀)-Italian (♂)) of *Tenebrio molitor* over a period of 56 days

Source			Adult survival (%)		Eggs per adult		Hatching rate (%)	
	Numerator df	Denominator df	F	P	F	P	F	P
Intercept	1	160	6865.9	< 0.001	1632.9	< 0.001	1596.7	< 0.001
Strain	3	160	7.5	< 0.001	1.4	0.234	5.1	0.002
Time (days)	7	160.000	62.2	< 0.001	65.2	< 0.001	159.1	< 0.001
Strain x Time (days)	21	160	0.8	0.657	1.9	0.015	2.1	0.005

**Fig. 2:**
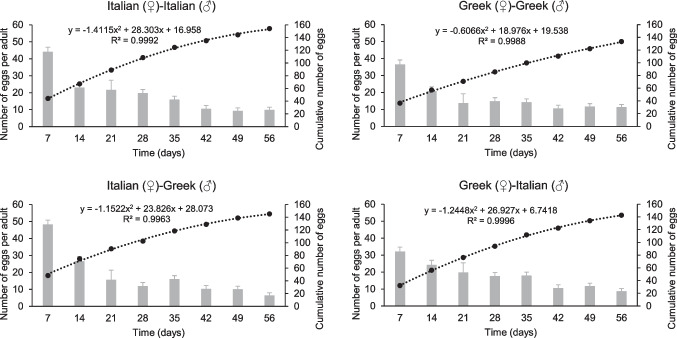
Oviposition every seven days of two inbred strains (Greek and Italian) and their outbred strains (Italian (♀)-Greek (♂) and Greek (♀)-Italian (♂)) of *Tenebrio molitor* over 56 days. The bars designate the number of eggs per adult at each time period. The dash lines reveal the fit curve for the cumulative number of eggs over time, and the dots symbolize the cumulative number of eggs at each time period. For dots, values depict means, while for bars, values depict means ± SE. Equations derived from the fit curve. The R.^2^ of the equation is also provided for each curve. For all inbred and outbred strains tested, n = 6

Regarding the cumulative number of eggs per adult at the end of the bioassay, no significant differences were recorded among the different inbred and outbred strains tested (F = 0.49, P = 0.68, df = 3.23), while the overall egg production per adult consistently decreased over time (Fig. 2). The highest cumulative number of eggs was produced by the inbred Italian strain (154 ± 28 eggs per adult), while the lowest cumulative number of eggs produced by the inbred Greek strain (133 ± 32 eggs per adult). Considering the egg production at specific intervals over time, the highest number of eggs per adult was detected 7 days after the onset of the bioassay for all inbred and outbred strains. For instance, at this interval (7 days), the egg production ranged between 32 ± 6 and 48 ± 7 eggs per adult. On the other hand, the lowest number of eggs per adult recorded at the end of the bioassay for all inbred and outbred strains. Specifically, 6 ± 3 to 11 ± 8 eggs per adult produced at the end of the bioassay (56 days). Considering the overall data, the number of eggs per adult was significantly affected by time (F = 65.2, P < 0.001), but it was not affected by strain (F = 1.4, P = 0.23) (Table 1).

**Fig. 3:**
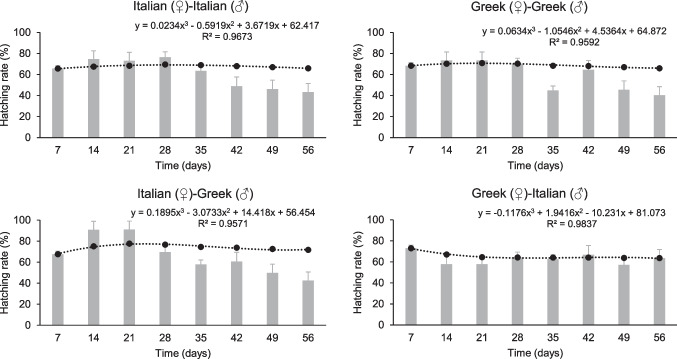
Hatching rate (%) of two inbred strains (Greek and Italian) and their outbred strains (Italian (♀)-Greek (♂) and Greek (♀)-Italian (♂)) of *Tenebrio molitor* over 56 days. The bars designate the larval hatching rate (%) at each time period. The dash lines reveal the fit curve for the cumulative hatching rate (%), and the dots symbolize the cumulative hatching rate at each time period. For dots, values depict means, while for bars, values depict means ± SE. Equations derived from the curve-fit. The R.^2^ of the equation is also provided for each curve. For all inbred and outbred strains, n = 6

Moreover, no significant differences were detected in terms of cumulative hatching rate at the end of the bioassay among inbred and outbred strains (F = 2.35, P = 0.10, df = 3.23), with rates ranging between 64% ± 7% and 72% ± 4%, at the end of the bioassay (Fig. 3). However, noticeable fluctuations detected during the entire observation period for all inbred and outbred strains (Fig. 3). Hence, the outbred Greek (♀)-Italian (♂) strain hatching rate fluctuated between 60 and 70% during the whole bioassay period. Moreover, for the outbred Italian (♀)-Greek (♂) strain, hatching rate fluctuations were more apparent, reaching from 90 to 40% until the end of the bioassay. For the inbred strains hatching rate fluctuated between 70 and 40%. Based on the linear mixed model analysis, both factors, strain (F = 5.1, P = 0.002) and time (F = 159.1, P < 0.001), significantly affected the hatching rate (Table 1).

### Bioassay II

**Fig. 4:**
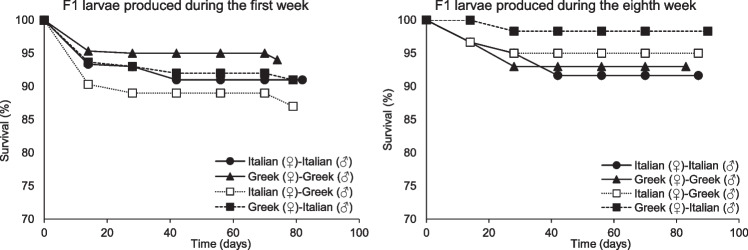
Survival rate (%) of larvae of two inbred strains (Greek and Italian) and their outbred strains (Italian (♀)-Greek (♂) and Greek (♀)-Italian (♂)) of *Tenebrio molitor* produced during the first and the eighth week after adults’ mating (For the larvae produced on the first week after adults’ mating n = 6, while for the larvae produced on the eighth week after adults’ mating n = 4)

The larval survival rate was high until the end of the bioassay (> 87%) for all inbred and outbred strains for the larvae produced during the first week after parental mating (Fig. 4). In this context, the highest rate of larval survival was recorded for the inbred Greek strain (94% ± 3%), while the lowest rate of larval survival recorded for the outbred Italian (♀)-Greek (♂) strain (87% ± 2%). For the larvae produced during the eighth week after parental mating, the survival was also high (> 92%) for all inbred and outbred strains (Fig. 4). The highest survival rate observed for the outbred Greek (♀)-Italian (♂) strain (98% ± 3%). Regarding the survival rate for the larvae produced during the first week after parental mating, insignificant contrasts were detected among the tested strains. Similarly, the larvae produced during the eighth week after parental mating followed a similar pattern concerning their survival rate. In contrast, significant differences detected for the outbred strains; Italian (♀)-Greek (♂) strain (F = 8.1, P = 0.02, df = 1.9) as well as for Greek (♀)-Italian (♂) strain (F = 9.0, P = 0.02, df = 1.9) among larvae produced from parental adults of different age (i.e., during the first and the eighth week after adult’s mating).

**Fig. 5:**
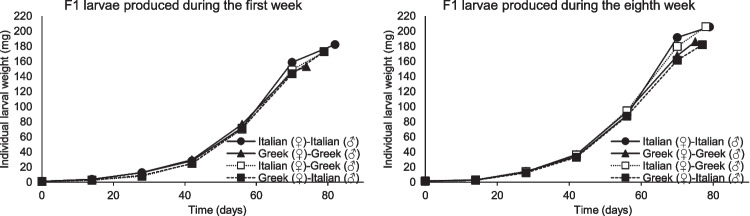
The average weight of two inbred strains (Greek and Italian) and their outbred strains (Italian (♀)-Greek (♂) and Greek (♀)-Italian (♂)) of *Tenebrio molitor* produced during the first and the eighth week after adults’ mating (For the larvae produced on the first week after adults’ mating n = 6, while for the larvae produced on the eighth week after adults’ mating n = 4)

Concerning the final weight of larvae produced during the first week after parental mating, significant differences were recorded among the different strains tested (F = 13.13, P < 0.001, df = 3.23). The final average larval weight for all inbred and outbred strains ranged between 145 ± 17 and 182 ± 7 mg (Fig. 5). Among the strains tested, the lowest and the highest final larval weight recorded for the inbred Greek strain and the inbred Italian strain, respectively. The outbred strains provided similar results, gaining a final larval weight between 172 ± 7 and 173 ± 11 mg. During the eighth week after parental mating, the larvae produced gained a final larval weight between 181 ± 10 and 206 ± 11 mg (Fig. 5). The lowest final larval weight recorded for the outbred strain Greek (♀)-Italian (♂) and the inbred Greek strain (181 ± 10 and 186 ± 21 mg, respectively). The highest final weights calculated for the inbred Italian strain as well as for the outbred Italian (♀)-Greek (♂) strain (205 ± 21 mg). Nevertheless, no statistical differences were detected for the final larval weight of the larvae produced during the eighth week after parental mating among the different strains tested (F = 2.32, P = 0.13, df = 3.15). However, between larvae produced by adult parents of different ages (i.e., during the first and the eighth week after parental mating), significant differences were recorded for the inbred Italian (F = 6.2, P = 0.03, df = 1.9) and Greek (F = 11.1, P = 0.01, df = 1.9) strain, as well as for the outbred Italian (♀)-Greek (♂) strain (F = 35.9, P < 0.001, df = 1.9) while, for the outbred Greek (♀)-Italian (♂) no statistical differences recorded (F = 1.7, P = 0.22, df = 1.9). Interestingly, the highest larval weight recorded for the larvae produced during the eighth week after parental mating for all inbred and outbred strains tested.

**Fig. 6:**
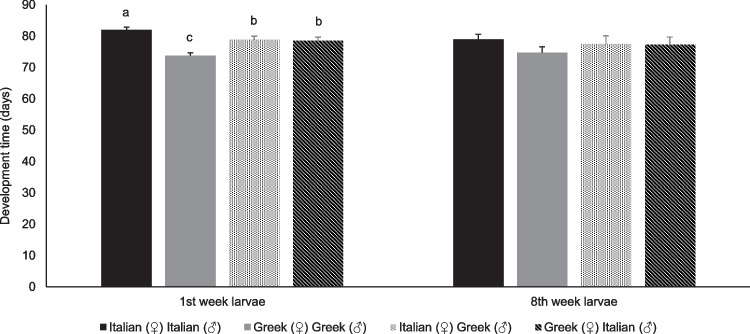
Developmental time (days) of larvae of two inbred strains (Greek and Italian) and their outbred strains (Italian (♀)-Greek (♂) and Greek (♀)-Italian (♂)) of *Tenebrio molitor* produced during the first and the eighth week after adults’ mating (n = 6). Lowercase letters depict significant differences between larvae produced from adults of different strains for the same time interval, while the absence of letters depicts no significant differences. However, for each time interval means with the different lowercase letter, differ significantly. For each case, values designate means ± SEM (For the larvae produced during the first week after adults' mating, n = 6, while for the larvae produced during the eighth week after adults' mating, n = 4)

Concerning the larvae produced during the first week after parental mating, development time ranged between 74 ± 0.8 and 82 ± 0.9 days for all strains, while the respective development time for the larvae produced during the eighth week after parental mating ranged between 75 ± 1.8 and 79 ± 1.6 days (Fig. 6). The development time of larvae during the first week after parental mating they were significantly influenced by strain (Mantel-Cox χ^2^ = 20.4, P < 0.001, df = 3), while no significant differences recorded between inbred and outbred strains for larvae produced during the eighth week after parental mating.

## Discussion

Commercial insect producers and research institutions aim to reveal insects’ desired characteristics to improve the fast-growing sector of insect mass-rearing. A common way to improve fitness-related traits is cross-breeding among insect strains (Eriksson and Picard 2021). In the general context of artificial selection and selective breeding for *T. molitor*, a few studies consider improving important economic traits such as insects’ body size, weight and growth rate, fecundity, and feed conversion efficiency (Leclercq 1963; Morales-Ramos et al. 2019). Past and recent research reveals that the selection of specific strains may also be beneficial for the growth and development of *T. molitor* larvae, as well as offspring production and survival (Adamaki-Sotiraki et al. 2022a, b; Rumbos et al. 2021; Urs and Hopkins 1973b, a). Based on the aforementioned reported differences in larval fitness traits and adults' performance, it is evident that insect farming systems' production will be optimized by selecting the proper strain. However, our data indicate a gap in terms of the cross-breeding effect among different strains of *T. molitor* on adults' performance and their offspring's fitness characteristics, especially at the larval stage.

Our study is the first one to assess the cross-breeding of two strains of *T. molitor*. Our results indicate good mating compatibility among adults of the different strains tested. Good mating compatibility is evident from the high reproductive output and the hatching rates over time, both for inbred and outbred strains. In general, data on the evaluation of cross-breeding of different insect strains are scarce and usually oriented towards life table characteristics of the silkworm, *B. mori*, and the honeybee, *Apis mellifera* (L.) (Hymenoptera: Apidae), as well as specific biocontrol agents (Eriksson and Picard 2021). Several studies related to sericulture have shown that cross-breeding has been a valuable tool in creating new and productive silkworm strains (Doddaswamy et al. 2009; Neshagaran Hemmatabadi et al. 2016). For instance, Reddy et al (2010) reported that the outbred crosses of divergent geographic eco races of *B. mori* revealed high heterosis potential and good compatibility of parents for yield contributing traits; fecundity, shell weight, and silk ratio. Along the same lines, Doddaswamy et al (2009), while experimenting with inbred and outbred crosses of *B. mori*, reported that F1 hybrids of outbred crosses performed better in terms of specific economic traits, such as tightly formed shells of spun cocoons, uniform growth, and consistent crop performance, compared to inbred crosses. Similarly, Bhargava et al (1995) tested the cross-breeding of five *B. mori* races and reported differences for seven silk technological characters; the weight of cocoon and shell, cocoon shell ratio, percentage of raw silk, length of silk filament, silk reliability, as well as silk neatness. However, cross-breeding only sometimes results in improving all economically valuable traits. For instance, Sohn and Ramirez (1999) studied the single, three-way, and double cross hybrids from four parental lines of *B. mori* in terms of cocoon quality and reported the correlation between cocoon weight and the presence of silken shell of cocoon was negative. Although from another perspective, that of silk production, the studies mentioned above show that cross-breeding can be a precious tool in developing high-quality *B. mori* hybrids. Apart from being a primary producer of silk, this species recently approved in the EU as an ingredient of poultry and pig diets, as well as aquafeeds (EU Regulation 2021/1925 (EC 2021c). However, no studies have been published regarding the effect of cross-breeding on the growth parameters of *B. mori* as an edible insect. We did not detect any apparent heterosis effect for the outbred strains tested, as offspring performance, quantified as larval survival and final larval weight, followed a similar pattern among the crossings examined in our study. In one of the few studies on *T. molitor* crosses, the crossing of two strains with different lengths, i.e., a small and a large strain, resulted in individuals of the F1 generation with an intermediary size (Schuurman 2013). Therefore, it becomes evident that classical breeding, in which genes are rearranged randomly, does not always result in offspring with improved characteristics. However, the recent access to the entire *T. molitor* genome opens new opportunities for genetic breeding and substantially contributes to producing new *T. molitor* lines with desirable traits (Eleftheriou et al. 2021).

Focusing on adult survival, egg production, and hatching rate, the inbred Italian strain and the outbred Italian (♀)-Greek (♂) strain gave the best results, even though no significant differences recorded between the strains tested. Indicatively, the outbred Italian (♀)-Greek (♂) strain produced the highest cumulative number of eggs, while it came up with the highest cumulative hatching rate. In accordance with the conclusions of McNamara et al (2009), who stated that organisms might choose to invest more in reproduction while they are still young, adult fecundity in our study declined while adults were getting older for inbred and outbred strains. The highest number of eggs oviposited 7 days after the onset of the bioassay. According to Morales-Ramos et al (2012), the highest rates of reproduction (33 eggs per female per week) takes place early in life for *T. molitor*, i.e., within the first two to three weeks of adults’ life. However, Adamaki-Sotiraki et al (2022b) reported that there might be a strain effect on the exact time that beetles reach their highest reproductive output. Interestingly, in our study, the exact time might have been affected by female parents of the different strains. For instance, for the inbred Italian strain and the outbred Italian (♀)-Greek (♂) strain, the highest reproductive output occurred at 28 days, while for the inbred Greek strain and the outbred Greek (♀)-Italian (♂) strain at 35 days. Based on our data, fluctuations in egg production were not vigorously expressed in our study, and this may be due to the mesh that was utilized in order to separate the adults from their offspring. We hypothesize that adding this mesh will likely minimize cannibalism, which is very common in *T. molitor* (Berggreen et al. 2018; Gerber and Sabourin 1984; Halliday et al. 2015). However, in another study, Adamaki-Sotiraki et al (2022b) did not use a mesh to separate adults from their offspring and reported that the fluctuations in egg production were due to the proneness of adults of *T. molitor* to cannibalize their eggs. The female adult beetles used in our study were not labeled/colored. Consequently, whether female survival has affected the number of eggs produced could be argued with certainty. Nevertheless, based on the existing literature, the lifespan of *T. molitor* adults is normally not influenced by sex (Rho and Lee 2016).

The present study's data may also indicate that when female adults from the Italian strain are involved, better offspring, in terms of larval weight, are produced. For instance, larvae produced during the eighth week after mating for the inbred Italian strain and the outbred Italian (♀)-Greek (♂) strain cluster together, gaining the highest larval weight, while the inbred Greek strain and the outbred Greek (♀)-Italian (♂) strain gained a similar larval weight. Additionally, comparing weights recorded for larvae produced during the first week after parental mating with the weights of larvae produced during the eighth week after parental mating, it is apparent that older parents tend to produce larvae with higher weight. Although from another point of view, that of a stored-product insect, several studies have explored the effect of the parental age on the offspring fitness for *T. molitor* (Howe 1956; Ludwig and Fiore 1960; Tracey 1958). For instance, Ludwig and Fiore (1960) studied the correlation of parental age with the lifecycle of *T. molitor* larvae and reported that the increase in the growth rate of larvae was associated with an increase in parental age, while larvae from older parents also gained more weight. Similar results were also reported by Howe (1956), who found that larvae that originated from old *T. molitor* females developed faster than those that originated from young females. In addition, Tracey (1958) supported that larvae from older parents gain weight faster at an earlier age than larvae produced from younger parents, which is also evident in the results of our study. However, what should also pointed out is that, in our study, due to the lower availability of larvae, a lower larval density (15 instead of 50 individuals per vial) was used in the bioassay with larvae produced during the eighth week after adults’ mating compared to the bioassay with larvae produced during the first week. Consequently, the differences mentioned above could also be attributed to the different densities rather than other biological parameters. Finally, the compatibility of the different strains tested here can be considered high, considering the high larval survival rates recorded in the outbred strains.

## Conclusions

The current study urges to take the strain effect on *T*. *molitor* fitness traits one step further, underlining the technique of cross-breeding of insect strains originating from different geographical areas and its utilization towards the production of new *T. molitor* lines with desirable characteristics. Our results indicate that strain-specific characteristics (i.e., larval weight) can be optimized by cross-breeding with an additional strain. In addition, even if, in some of the combinations examined here, the differences in larval weight were marginal, cross-breeding effects may be remarkable if translated at a commercial scale. Hence, this cross-breeding effort may contribute to the optimization of currently adopted rearing protocols, utilizing the divergent phenotypic characteristics of strains that provide specific traits to their offspring, heading off the generic mass-rearing procedure for *T*. *molitor*. Further research on phenotype-genotype relationships could be utilized to lead us to more optimal selective breeding approaches between different strains.

## Data Availability

Not applicable.
